# The Effect of Intraoperative Sounds of Saw and Hammer on Psychological Condition in Patients with Total Knee Arthroplasty: Prospective Randomized Study

**DOI:** 10.1155/2015/690569

**Published:** 2015-02-22

**Authors:** Erkam Kömürcü, Hasan Ali Kiraz, Burak Kaymaz, Umut Hatay Gölge, Gürdal Nusran, Ferdi Göksel, Hasan Şahin, Dilek Ömür, Volkan Hancı

**Affiliations:** ^1^Department of Orthopaedics and Traumatology, Çanakkale Onsekiz Mart University Faculty of Medicine, 17100 Çanakkale, Turkey; ^2^Department of Anesthesiology and Reanimation, Çanakkale Onsekiz Mart University Faculty of Medicine, 17100 Çanakkale, Turkey; ^3^Department of Anesthesiology and Reanimation, Dokuz Eylül University Faculty of Medicine, 35340 İzmir, Turkey

## Abstract

*Purpose*. Surgical procedures are likely to be stressful for patients and their families. Total knee arthroplasty (TKA) is a major surgical procedure used in the treatment of osteoarthritis. During this procedure the sounds of the saw and hammer may irritate the patient and adversely affect mood. The present study examines the effect of these intraoperative sounds during TKA on postoperative mood and anxiety, by comparing two different anesthetic procedures. *Methods*. A total of 40 patients who underwent TKA for grade IV gonarthrosis participated in the study. Patients were randomly divided into two groups: 20 patients in the general anesthesia group and 20 patients in the spinal anesthesia group. Mood and anxiety changes were evaluated using the Profile of Mood States (POMS) and State-Trait Anxiety Inventory (STAI) instruments, respectively. *Results*. The postoperative POMS value in the spinal anesthesia group was definitively higher than the general anesthesia group, though the difference in preoperative and postoperative POMS and STAI scores between the two groups was not significant. *Conclusion*. It would seem that sounds of hammer and saw have no evident negative effect on patient's mood. Regional anesthesia is advisable for TKA patients and appropriate sedation can be administered during the operation if needed.

## 1. Introduction

From beginning to end, surgical procedures can be very stressful for patients and for their families. Patients endure subjective feelings of risk and stress in order to regain their health. This may lead to high levels of anxiety in both the preoperative and postoperative periods [1]. These psychological problems may present as physical symptoms including headache, nausea, or confusion and may obscure the clinical tableau [2]. In relation to perception of pain anxiety in the preoperative and perioperative period, or presenting in the postoperative period, previous studies have shown that anxiety levels may lower the pain threshold, alter perceptions of pain, and increase postoperative analgesic consumption [3].

Total knee arthroplasty (TKA) is a commonly applied elective surgery which aims to enhance quality of life. The objective is to reduce pain and increase function. For a successful outcome, significant factors may include appropriate patient choice, careful and effective surgical procedure, and adequate rehabilitation [4]. For successful rehabilitation the level of patients' mood and anxiety is also significant [5]. Anxiety reduces patients' physical and emotional energy, causing fatigue and negatively affecting the healing process [6, 7]. In addition, it is ironic that TKA ranks first for noises, such as the sound of saws and hammers, which may alarm patients during the perioperative period. Many studies indicate that the highest anxiety is experienced by patients in the period before surgery [1]. However, there are relatively few studies of mood changes in orthopedic patients during and after operations [6–8].

Previous studies have examined the effects of postoperative anxiety on patients observing their arthroscopic operations in real time. For operations with low noise levels, like arthroscopy, watching the procedure on live video has been determined to reduce patient's anxiety [9]. Another study reports that regional anesthetic administration changes postoperative mood [10]. Studies on this topic are controversial and for procedures with intense ambient noise, such as TKA, there are to date no studies evaluating effects on mood and anxiety.

There are a number of approaches toward testing of mood and anxiety. While the Profile of Mood States (POMS) is a psychological rating scale used to assess transient, distinct mood states, the State-Trait Anxiety Inventory (STAI) measures and discriminates two types of anxiety—state anxiety and trait anxiety [11, 12]. We used POMS and STAI (both state and trait forms) to measure mood and anxiety of the patients, respectively. POMS and STAI were used in many similar studies [13–19].

The present study investigates the impact of the surgical environment, especially the sounds of saw and hammer in the operating room, on patient's mood and anxiety after the operation. The design randomly divides patients into two groups: spinal anesthesia and general anesthesia groups and the hypothesis of this study is that regional anesthesia, administered without accompanying sedation, will not negatively affect the mood and anxiety in the postoperative period.

## 2. Materials and Methods

This study has received approval from the Institutional Ethics Committee and was conducted in accordance with the Declaration of Helsinki. Each participant received oral and written information about the study and signed a consent form.

Forty patients with planned elective TKA, and ranging in age from 55 to 80 years, participated in the study. Sampling criteria included advanced-stage gonarthrosis (Kellgren-Lawrence classification grade IV gonarthrosis), willingness to participate, and education to at least middle school standard [20]. Patients with previous hip and/or knee arthroplasty operations, known psychological disorders, or literacy problems were not included in the study. Patients were not given premedication before the procedure, and the Verbal Numeric Scale (VNS) was explained following the sequential order of sealed envelopes. In performing VNS, we asked the patient the following: “On a scale of 0 to 10, with 0 being “no pain” and 10 being “the most intense pain imaginable,” how would you rate the severity of your pain right now?” The number verbalized by the patient is considered as VNS score [21]. There were 20 patients in each group.

Before the procedure each patient's basal hemodynamic parameters were recorded. The hemodynamic markers of heart rate (HR), systolic arterial pressure (SAP), diastolic arterial pressure (DAP), mean arterial pressure (MAP), and SpO_2_ values were recorded throughout surgery.

In the spinal anesthesia group, patients were administered spinal anesthesia while held in a sitting position by an assistant, in the L3-4 or L4-5 interspinous interval. Spinal anesthesia was administered using 2.5 mL of hyperbaric bupivacaine (12.5 mg). When the sensory block level reached T8, the operating table was brought to a neutral position, and permission was given to start the operation. Control values for MAP falling by more than 20% or below 60 mm Hg were evaluated as hypotension, and fast fluid replacement (50 mL/min) was initiated. If no response was seen within 3 minutes, treatment was IV bolus of 5 mg ephedrine. HR less than 50 bpm was evaluated as bradycardia, and IV bolus of 0.5 mg atropine was administered for treatment.

In the total intravenous anesthesia (general anesthesia) group, patients were given 1 *μ*g/kg remifentanil for 30 to 60 seconds, IV bolus for induction, and 0.05 to 0.25 *μ*g/kg/min infusion after anesthesia deepened or shallowed. In the 2 minutes after administration of propofol 1.5 mg/kg and vecuronium bromide 0.1 mg/kg IV, endotracheal intubation was completed after 100% oxygen ventilation through a mask. For maintenance, 4 to 6 mg/kg/hr propofol infusion was initiated. Mechanical ventilation with tidal volume of 8 mL/kg, respiration count of 10 breaths per minute, 50% O_2_, and 50% air was administered. MAP falling by more than 20% or below 60 mmHg was evaluated as hypotension, and fast fluid replacement (50 mL/min) was initiated. If the patient did not respond, the remifentanil dose was reduced by 50%. If levels did not improve, the speed of propofol infusion was reduced by 50%. If no improvement was seen within three minutes, treatment was IV bolus of 5 mg ephedrine. HR below 50 bpm was evaluated as bradycardia and IV bolus of 0.5 mg atropine was administered for treatment.

During surgery all patients were monitored for side effects: blood and blood product transfusions, any complications, and total surgical duration were recorded. After operation, 2 L/min oxygen therapy was administered with mask to prevent postoperative respiratory depression, heating was administered to prevent shivering, and antiemetic therapy was administered to prevent nausea and vomiting in all patients if required. In the recovery room HR, SAP, DAP, MAP, and SpO_2_ monitoring parameters were recorded every 10 minutes. After eliminating the risk of early postoperative complications, patients with normal values for at least 60 minutes were sent to the orthopedic service. While the same surgeon performed all surgical procedures, exposure to the same brand and system of prosthetic was ensured.

The State-Trait Anxiety Inventory can be used in clinical settings to diagnose anxiety and to distinguish it from depressive syndromes. The inventory contains separate self-report scales to measure two distinct anxiety concepts: state anxiety (A-State) and trait anxiety (A-Trait). The STAI consists of two scales containing 20 items of 4 options each on a standard Likert scale coded from 0 to 3. The total score indicates which type of anxiety is prevalent [12]. POMS is a self-rating scale which consisted of 65 adjectives that were rated on a 5-point scale. POMS measures 6 identifiable mood or affective states: “tension-anxiety,” “depression-dejection,” “anger-hostility,” “vigor-activity,” “fatigue-inertia,” and “confusion-bewilderment.” To obtain a score for each mood factor, the sum of the responses is obtained for the adjectives defining the mood factor. A seventh score of total mood disturbance is also calculated by subtracting the score on the one positively scored subscale, vigoractivity, from the sum of the other five subscales [22].

The patients completed forms in the preoperative period and in the twelfth hour after operation for psychometric evaluation using the POMS [11] and STAI [12]. Also patient and doctor satisfactions were evaluated by the test of satisfaction that measured the satisfaction from grade 0 to grade 4 (0: not satisfied–4: fully satisfied).

## 3. Statistical Methods

Statistical analysis was performed with the Statistical Package for Social Sciences version 19.0 (IBM Corp. Released 2010. IBM SPSS Statistics for Windows, Version 19.0. Armonk, NY: IBM Corp.). Descriptive statistics were used to define sociodemographic and basic clinical variables. The chi-square test was used to compare the categorical variables. Normality of continuous data was determined by the Kolmogorov-Smirnov test. When normality of the distribution of variables was acceptable, Student's *t*-test and the paired samples *t*-test were used to analyze differences in scale between preoperative and postoperative data. Differences were considered significant at *P* < 0.05 for all tests (two-tailed).

## 4. Results

The demographic characteristics of the 40 patients (28 female, 12 male) included in the study showed no statistical difference between the two groups and had a homogeneous distribution (Table 1).

The anesthetic parameters and hemodynamic findings of both groups are shown in Table 2. In relation to anesthesia and surgical procedure duration, there was no significant statistical difference between the groups. In the general anesthesia and spinal anesthesia groups the average fluids given were 2100 ± 536.6 mL and 2512.0 ± 642.6 mL, respectively (*P* = 0.04). The heart rate in the spinal anesthesia group before and during the operation was higher, and this was also statistically significant (*P* < 0.05) (Table 2).

The required blood transfusions in the general and spinal anesthesia patients were 1.9 ± 0.6 and 2.1 ± 0.5 units, respectively (*P* = 0.378, independent samples test, *P* < 0.050). No difference was found between the groups in relation to blood transfusions.

In the postoperative period, shivering severe enough to cause discomfort to the patient occurred in three patients in the general anesthesia (7.5%) and one patient in the spinal anesthesia group (2.5%). In the postoperative period, nausea and respiratory depression were observed in three patients (7.5%) in the general group and one patient (2.5%) in the spinal anesthesia group. In both the perioperative and postoperative periods no additional complications were observed.

In general and spinal anesthesia administration, the preoperative and postoperative POMS and STAI measurement values showed no statistically significant difference (*P* = 0.179 and *P* = 0.893, resp., for general anesthesia; *P* = 0.266 and *P* = 0.203 for spinal anesthesia, paired samples *t*-test, *P* < 0.05). But in the postoperative period, the POMS value in the spinal anesthesia group was definitively higher than the general anesthesia group, although statistically not significant (Table 3).

According to genders, when the change in postoperative and preoperative POMS is investigated, the averages were −7.8 ± 29.2 in men and 2.9 ± 24.9 in women, and these were not statistically significant (*P* = 0.251, independent samples test, *P* < 0.05). The changes in postoperative and preoperative STAI averages for men and women were 3.5 ± 12.4 and 0.8 ± 15.6, respectively, and again this was not statistically significant (*P* = 0.597, independent samples test, *P* < 0.05).

Both general and spinal anesthesia patients showed a statistically significant correlation between preoperative and postoperative POMS results (*r* = 0.536, *P* = 0.015 and *r* = 0.656, *P* = 0.002, resp., paired samples *t*-test, *P* < 0.05), but there was no correlation between STAI results (*r* = 0.157, *P* = 0.509 and *r* = 0.261, *P* = 0.266, resp., paired samples *t*-test, *P* < 0.05).

While patients with operations completed with spinal anesthesia appeared to be more satisfied (*P* = 0.09, chi-square test, *P* < 0.05), there was no difference between general and spinal anesthesia groups in terms of doctor satisfaction (*P* = 0.537, chi-square test, *P* < 0.05).

## 5. Discussion

Total knee arthroplasty (TKA) is an effective treatment method for advanced knee osteoarthritis patients and is becoming more common. During TKA surgery, the sound of the cutting motor used for femoral and tibial osteotomy, the hammering sounds during placement of the prosthesis, and even the smell of cement can make this a frightening and stress-inducing situation for patients. There is cause for concern that this exposure to stress in the operating room may adversely affect a patient's emotional state in the postoperative period. To our knowledge, the effect of these sounds on mood states in patients undergoing TKA has not previously been studied.

Factors including youth, low educational attainment, and no prior experience of surgical intervention are predictors of higher subjective anxiety in the period before surgery [2, 23]. Studies by Nickinson and Szekely determined that, in the postoperative period, mood is more likely to be affected in women than in men [6, 24]. The present study, however, finds no difference in mood between the genders in preoperative and postoperative periods.

It is known that patients who have previously had hip or knee arthroplasty are more prone to depression in the postoperative period of the second surgery, by comparison with patients having their first arthroplasty operation [6]. For this reason the present study does not include patients who had previously undergone hip and knee arthroplasty operations.

Karanci and Dirik predicted that preoperative anxiety will show a significant decrease when measured in the postoperative period [2]. The present study, however, finds no significant change in mood and anxiety between the preoperative and postoperative periods. Contrary to the findings that postoperative distress levels may be predicted by preoperative distress levels [25, 26], the present study finds that preoperative and postoperative anxiety levels are not correlated and postoperative anxiety level could not be predicted by assessing the preoperative anxiety level. On the other hand, mood state showed a correlation between preoperative and postoperative periods in both groups. This may relate to the more dynamic and variable nature of anxiety itself and the possible impact of environmental conditions; mood changes do appear to be more stable.

Regional anesthesia methods are often the preferred choice for orthopedic surgery. This form of anesthesia reduces perioperative bleeding, postoperative embolic complications, postoperative nausea and vomiting, risk of aspiration, and postoperative pain. It has also been found to make rehabilitation easier. Disadvantages of spinal anesthesia include slower onset, spinal headaches, and micturition dysfunction [27–29].

Tanaka et al. asked patients to watch live video of their arthroscopic surgery and reported that these patients had greater levels of satisfaction after surgery [25]. Similarly, just as the cold and stressful environment of the operating room increased both perioperative and postoperative anxiety in patients, they concluded that, in the perioperative period, distracting interventions reduced patients' anxiety and improved postoperative results. These distractions included watching live video of the arthroscopy (non-TKA) and listening to music [1, 3, 30]. Bayar et al. found that the patients with spinal anesthesia watching their images of arthroscopic operation had lower postoperative STAI scores and visual feedback was effective in reducing anxiety, in both the perioperative and postoperative periods [9]. Adversely, in another study investigating the effect on mood of spinal anesthesia and laryngeal mask anesthesia in 46 patients undergoing hemorrhoidectomy, the evidence suggests that mood in the spinal anesthesia group disimproved in the postoperative period [10].

In the present study it was aimed to investigate the effects of intraoperative sounds of hammer and saw on the patients' mood and anxiety. Although it can be thought that the sounds of hammer and saw used in arthroplasty operations may be frightening and stress inducing, the preoperative and postoperative POMS and STAI measurement values showed no statistically significant difference in the present study. POMS value in the spinal anesthesia group was definitively higher than the general anesthesia group. Also patients with operations completed with spinal anesthesia appeared to be more satisfied. In light of this, it would seem that sounds of hammer and saw have no evident negative effect on patient's mood. One possible explanation for the satisfaction might be that spinal anesthesia lessens postoperative pain, allowing the early postoperative period, when pain is most strongly felt, to pass painlessly.

The present study also found differences between groups in the relation between anesthetic parameters of total fluid amounts given and heart rate. We consider that similarity on other parameters did not affect anxiety and mood data. The higher heart rate in the spinal anesthesia group may not be due to anxiety but due to the higher amount of fluids given to spinal anesthesia patients.

There is considerable evidence that anxiety and depression in patients adversely affect outcomes of surgery and increase length of hospital stay [6, 31, 32]. High levels of preoperative and perioperative anxiety and depression may cause serious pain or phantom pain and impact seriously on postoperative results [33, 34]. Mossey et al. found a relationship between the quality of patients' recovery and postoperative mood and also found that postoperative anxiety and depression adversely affected functional recovery [35].

In our view, mood during the postoperative period is a factor in achieving a successful outcome. TKA is an elective surgical procedure intended to enhance the patient's daily quality of life. In light of this, we believe that any factor that can be shown to affect postoperative results must be considered important.

In conclusion, it would seem that sounds of hammer and saw have no evident negative effect on patient's mood and in operations performed with spinal anesthesia, the patients were found to be more satisfied so that, with known advantages, regional anesthesia is advisable for TKA patients and appropriate sedation can be administered during the operation if needed.

## Figures and Tables

**Table 1:** Demographical parameters of all patients.

	General anesthesia	Spinal anesthesia	*x* ^2^/*t*	*P*
	(Group 1)	(Group 2)		
	*n* = 20	*n* = 20		
Women (*n*/%)	13/32.5	15/37.5	0.476	0.49^*^
Men (*n*/%)	7/17.5	5/12.5	*0.476 *	*0.49* ^*^
Age (M ± SD)	65.6 ± 7.2	65.2 ± 5.6	*0.197 *	*0.85* ^ #^
Weight (M ± SD) kg	86.9 ± 14.9	82.8 ± 13.1	*0.948 *	*0.35* ^ #^
Height (M ± SD) cm	161.3 ± 8.4	159.1 ± 5.9	*0.956 *	*0.35* ^ #^
ASA-score (M ± SD)	2 ± 0.3	2.2 ± 0.4	*−1.710 *	*0.96* ^ #^

^*^Chi-square test, ^#^independent samples test, *P* < 0.05, ASA-score: American Society of Anesthesiologists' score, M: mean, and SD: standard deviation.

**Table 2:** Comparison of anesthetic parameters.

	General anesthesia	Spinal anesthesia	*t*	*P*
	(Group 1)	(Group 2)		
	*n* = 20	*n* = 20		
Duration of anesthesia (min)	157.0 ± 30.0	156.1 ± 30.5	0.089	0.93^*^
Duration of surgery (min)	123.1 ± 26.1	126.6 ± 30.1	−0.393	0.70^*^
Total iv fluid (mL)	2100.0 ± 563.6	2515.0 ± 642.6	−2.171	0.04^*^
HR (pre-op)	74.4 ± 10.6	83.9 ± 13.0	−2.516	0.02^*^
HR (1. min)	70.8 ± 10.3	83.2 ± 12.3	−3.475	<0.01^*^
HR (30. min)	62.8 ± 8.2	75.3 ± 9.3	−4.525	<0.01^*^
HR (60. min)	65.7 ± 12.8	73.2 ± 2.0	−2.142	0.04^*^
HR (90. min)	64.2 ± 7.7	76.2 ± 5.9	−5.516	<0.01^*^
HR (120. min)	69.0 ± 10.4	78.0 ± 8.0	−2.400	0.03^*^
MAP (pre-op)	107.0 ± 18.8	107.8 ± 12.1	−0.150	0.88^*^
MAP (1 min)	92.0 ± 29.1	102.7 ± 15.0	−1.462	0.16^*^
MAP (30 min)	90.7 ± 17.5	94.1 ± 13.4	−0.691	0.49^*^
MAP (60 min)	94.3 ± 16.5	93.9 ± 12.6	0.86	0.93^*^
MAP (90 min)	90.9 ± 14.4	95.0 ± 15.8	−0.858	0.40^*^
MAP (120 min)	93.8 ± 22.0	100.2 ± 15.5	−0.825	0.42^*^
SpO_2_ (30 min)	99.1 ± 1.1	99.2 ± 0.7	−0.327	0.75^*^
SpO_2_ (60 min)	99.0 ± 1.2	99.0 ± 0.9	0.152	0.88^*^
SpO_2_ (90 min)	99.2 ± 0.9	99.3 ± 0.7	−0.380	0.71^*^
SpO_2_ (120 min)	99.1 ± 1.8	99.1 ± 0.6	0.027	0.98^*^

^*^Independent samples test, *P* < 0.05, HR: heart rate, MAP: mean arterial pressure, preop: preoperative, min: minute.

**Table 3:** Comparison of anesthetic procedures on psychological condition.

	General anesthesia	Spinal anesthesia	*t* value	*P* value
	(Group 1)	(Group 2)		
	*n* = 20	*n* = 20		
Preoperative POMS	63.6 ± 28.2	65.7 ± 33.9	*−0.208 *	*0.836* ^*^
Postoperative POMS	56.0 ± 20.2	72.7 ± 31.9	*−1.977 *	***0.055*** ^*^
Preoperative STAI	89.9 ± 12.3	90.7 ± 8.0	*−0.259 *	*0.797* ^*^
Postoperative STAI	89.4 ± 13.0	94.4 ± 12.0	*−1.276 *	*0.210* ^*^
Postop-pre-op POMS	−7.6 ± 24.4	7.0 ± 27.3	*−1.784 *	*0.082* ^*^
Postop-pre-op STAI	−0.5 ± 16.5	3.7 ± 12.5	*−0.908 *	*0.370* ^*^

^*^Independent samples test, *P* < 0.05.

## References

[1] Wang S., Kulkarni L., Dolev J., Kain Z. N. (2002). Music and preoperative anxiety: a randomized, controlled study. *Anesthesia and Analgesia*.

[2] Karanci A. N., Dirik G. (2003). Predictors of pre- and postoperative anxiety in emergency surgery patients. *Journal of Psychosomatic Research*.

[3] Chan Y. M., Lee P. W. H., Ng T. Y., Ngan H. Y. S., Wong L. C. (2003). The use of music to reduce anxiety for patients undergoing colposcopy: a randomized trial. *Gynecologic Oncology*.

[4] Ritter M. A., Gandolf V. S., Holston K. S. (1989). Continuous passive motion versus physical therapy in total knee arthroplasty. *Clinical Orthopaedics and Related Research*.

[5] Hampel P., Graef T., Krohn-Grimberghe B., Tlach L. (2009). Effects of gender and cognitive-behavioral management of depressive symptoms on rehabilitation outcome among inpatient orthopedic patients with chronic low back pain: a 1 year longitudinal study. *European Spine Journal*.

[6] Nickinson R. S. J., Board T. N., Kay P. R. (2009). Post-operative anxiety and depression levels in orthopaedic surgery: a study of 56 patients undergoing hip or knee arthroplasty. *Journal of Evaluation in Clinical Practice*.

[7] Salamon E., Kim M., Beaulieu J., Stefano G. B. (2003). Sound therapy induced relaxation: down regulating stress processes and pathologies. *Medical Science Monitor*.

[8] Salazar F., Doñte M., Boget T. (2011). Intraoperative warming and post-operative cognitive dysfunction after total knee replacement. *Acta Anaesthesiologica Scandinavica*.

[9] Bayar A., Tuncay I., Atasoy N., Ayoğlu H., Keser S., Ege A. (2008). The effect of watching live arthroscopic views on postoperative anxiety of patients. *Knee Surgery, Sports Traumatology, Arthroscopy*.

[10] Kisli E., Agargun M. Y., Tekin M., Selvi Y., Karaayvaz M. (2007). Effects of spinal anesthesia and laryngeal mask anesthesia on mood states during hemorrhoidectomy. *Advances in Therapy*.

[11] McNair D. L. M., Droppleman L. (1992). *Profile of Mood States Manual*.

[12] Hidano N., Fukuhara M., Iwawaki M., Soga S., Spielberger C. D. (2000). *State-Trait Anxiety Inventory—Form JYZ*.

[13] Udani J. K. (2013). Effects of superulam on supporting concentration and mood: a randomized, double-blind, placebo-controlled crossover study. *Evidence-Based Complementary and Alternative Medicine*.

[14] Balsamo S., Diniz L. R., dos Santos-Neto L. L., da Mota L. M. (2014). Exercise and fatigue in rheumatoid arthritis. *Israel Medical Association Journal*.

[15] Maroufi A., Khomand P., Ahmadiani S., Alizadeh N. S., Gharibi F. (2014). Prevalence and quality of anxiety in patients with epilepsy. *Epilepsy & Behavior*.

[16] Kovacs Z., Szabo C., Fulop E. (2013). Therapy helps—psychosocial support for patients diagnosed with breast cancer, reducing anxiety and depression. *Psychiatria Hungarica*.

[17] Abacı F. B., Gökçe S., Tuygun N., Karacan C. D., Öner Ö. (2013). Psychosocial status and quality of life in mothers of infants with colic. *The Turkish Journal of Pediatrics*.

[18] Granziera E., Guglieri I., Del Bianco P. (2013). A multidisciplinary approach to improve preoperative understanding and reduce anxiety: a randomised study. *European Journal of Anaesthesiology*.

[19] Kim K. H., Woo H. Y., Lim S. W. (2008). Association study of a serotonin receptor 2A gene-1438A/G polymorphism and anxiety-related traits. *Psychiatry Investigation*.

[20] Kellgren J. H., Lawrence J. S. (1957). Radiological assessment of osteo-arthrosis. *Annals of the Rheumatic Diseases*.

[21] Young D. M., Mentes J. C., Titler M. G. (1999). Acute pain management protocol. *Journal of Gerontological Nursing*.

[22] Selvi Y., Gulec M., Aydin A., Besiroglu L. (2011). Psychometric evaluation of the Turkish language version of the profile of mood states (POMS). *Journal of Mood Disorders*.

[23] Mackenzie J. W. (1989). Daycase anaesthesia and anxiety. A study of anxiety profiles amongst patients attending a Day Bed Unit. *Anaesthesia*.

[24] Székely A., Benkö E., Varga A., Mészáros R. (2001). Postoperative depression after open heart surgery. *Orvosi hetilap*.

[25] Tanaka M., Takahashi T., Yamamoto H. (2003). Simultaneous live video presentation during knee arthroscopy. *Journal of Orthopaedic Science*.

[26] De Groot K. I., Boeke S., Van Den Berge H. J., Duivenvoorden H. J., Bonke B., Passchier J. (1997). The influence of psychological variables on postoperative anxiety and physical complaints in patients undergoing lumbar surgery. *Pain*.

[27] Somri M., Matter I., Parisinos C. A. (2012). The effect of combined spinal-epidural anesthesia versus general anesthesia on the recovery time of intestinal function in young infants undergoing intestinal surgery: a randomized, prospective, controlled trial. *Journal of Clinical Anesthesia*.

[28] Harsten A., Kehlet H., Toksvig-Larsen S. (2013). Recovery after total intravenous general anaesthesia or spinal anaesthesia for total knee arthroplasty: a randomized trial. *British Journal of Anaesthesia*.

[29] Salman C., Kayacan N., Ertuĝrul F., Bigat Z., Karsli B. (2013). Combined spinal-epidural anesthesia with epidural volume extension causes a higher level of block than single-shot spinal anesthesia. *Revista Brasileira de Anestesiologia*.

[30] Nilsson U., Rawal N., Unosson M. (2003). A comparison of intra-operative or postoperative exposure to music—a controlled trial of the effects on postoperative pain. *Anaesthesia*.

[31] Oxlad M., Stubberfield J., Stuklis R., Edwards J., Wade T. D. (2006). Psychological risk factors for increased post-operative length of hospital stay following coronary artery bypass graft surgery. *Journal of Behavioral Medicine*.

[32] Philips B. U., Bee D. E. (1980). Determinants of postoperative recovery in elective orthopedic surgery. *Social Science and Medicine A*.

[33] Richardson C., Glenn S., Horgan M., Nurmikko T. (2007). A prospective stu dy of factors associated with the presence of phantom limb pain six months after major lower limb amputation in patients with peripheral vascular disease. *Journal of Pain*.

[34] Riediger W., Doering S., Krismer M. (2010). Depression and somatisation influence the outcome of total hip replacement. *International Orthopaedics*.

[35] Mossey J. M., Mutran E., Knott K., Craik R. (1989). Determinants of recovery 12 months after hip fracture: the importance of psychosocial factors. *The American Journal of Public Health*.

